# Co-amoxicillin–induced reactivation of auto-brewery syndrome in a patient treated for community acquired pneumonia: a clinical case report

**DOI:** 10.1093/omcr/omag167

**Published:** 2026-09-08

**Authors:** Philipp Widmer, Flavia A Miesch, Barbara Scotti, Gian Erni, Roland Sperb

**Affiliations:** Department of Internal Medicine, Luzerner Kantonsspital, Spitalstrasse 16A, Sursee 6210, Switzerland; Department of General Surgery, Luzerner Kantonsspital, Spitalstrasse 16A, Sursee 6210, Switzerland; Department of Emergency Medicine, Luzerner Kantonsspital, Spitalstrasse 16A, Sursee 6210, Switzerland; Department of Emergency Medicine, Luzerner Kantonsspital, Spitalstrasse 16A, Sursee 6210, Switzerland; Department of Internal Medicine, Luzerner Kantonsspital, Spitalstrasse 16A, Sursee 6210, Switzerland

**Keywords:** auto-brewery syndrome, gut fermentation syndrome, amoxicillin/clavulanic acid, Co-amoxicillin, antibiotic-induced Dysbiosis, acute alcohol intoxication

## Abstract

Auto-Brewery Syndrome (ABS) is a rare metabolic condition characterized by endogenous ethanol production through fermentation of ingested carbohydrates by intestinal microorganisms. Clinical manifestations resemble alcohol intoxication, including ataxia, dysarthria, gastrointestinal symptoms, and altered mental status, despite the absence of alcohol intake. We report a case of a patient in his mid-40s who developed ABS reactivation following treatment with Amoxicillin/Clavulanic acid for community-acquired pneumonia. The patient was successfully managed with targeted antimicrobial therapy, dietary carbohydrate restriction, and probiotic supplementation, resulting in clinical improvement. This case highlights a rare instance of antibiotic-associated ABS relapse and underscores the diagnostic challenge of this unrecognized microbial condition.

## Introduction

Auto-Brewery Syndrome (ABS), also known as Gut Fermentation Syndrome (GFS), is a rare and underrecognized medical condition in which intoxicating levels of ethanol are produced endogenously via fermentation of carbohydrates by yeast, fungi or bacteria in the gastrointestinal tract [1]. Patients with ABS may present with symptoms of alcohol intoxication, such as dizziness, disorientation, slurred speech, and altered behaviour, despite no alcohol consumption. Endogenous ethanol production is driven by microbial fermentation of carbohydrates, involving organisms such as *Candida albicans*, *Candida krusei*, Candida glabrata, *Saccharomyces cerevisiae*, and Klebsiella pneumonia [2, 3]. Recent studies have demonstrated that endogenous ethanol-producing gut bacteria are not only associated with ABS but also with metabolic liver diseases such as non-alcoholic fatty liver disease, highlighting the clinical relevance of microbial ethanol metabolism [4, 5]. Diagnosis of ABS begins with analysing medical history, focusing on signs of unexplained alcohol intoxication and denying alcohol consumption [6]. Further evaluation includes upper and lower endoscopy to collect secretions for fungal and bacterial culture [6]. A confirmatory diagnostic is the carbohydrate challenge test, where the patient consumes 200 g of glucose and Blood Alcohol Concentration (BAC) and Breath Alcohol Concentration (BrAC) are measured at intervals to detect ethanol production [7].

Treatment of acute alcohol intoxication involves supportive care, such as intravenous fluid and respiratory stabilisation, followed by longer-term intervention. This includes dietary modification, reducing carbohydrate and high protein intake. Antifungal or antibacterial therapy may be used to reduce responsible microbial overgrowth, and probiotics can help restore microbiota. The patient should also avoid ethanol and foods high in yeast or mold [7, 8]. ABS can significantly impair quality of life and may lead to social, legal, and medical consequences [7]. Data on ABS are scarce; small studies and case reports indicate it may be underdiagnosed. The lack of clear diagnostic guidelines and vague symptoms results in misdiagnosis by healthcare providers [8].

As a result, diagnosis is frequently delayed, leading to inadequate or late treatment. Increasing clinical awareness is essential for timely diagnosis and effective management. In this case report, we present a relapse of ABS following treatment of community-acquired pneumonia with Amoxicillin/Clavulanic acid, highlighting the potential role of antibiotic-induced dysbiosis in reactivating this condition.

## Case report

In early 2025, a man in his mid-40s was brought to the emergency room of a hospital in Switzerland after being found unresponsive at home. Upon arrival, vital parameters were stable, Glasgow Coma Scale (GCS) was 9 (E1, V2, M6). Arterial blood gas analysis was within normal limits. Given the reduced level of consciousness of unclear aetiology, empirical administration of naloxone and flumazenil was performed with no clinical improvement. A cranial CT scan showed no evidence of intracranial haemorrhage or structural brain damage. Laboratory findings were unremarkable except for a BAC of 43.6 mmol/l. The patient’s partner reported a known diagnosis of ABS and confirmed abstinence prior to admission. The general practitioner verified the diagnosis and noted that five days earlier, the patient was started on Amoxicillin/Clavulanic acid due to pneumonia.

His clinical history spans five years, including episodes of alcohol misuse in 2020 that resolved after inpatient psychiatric rehabilitation. Two months later, despite confirmed abstinence, he was hospitalized for psychological reasons with a BAC of 46.8 mmol/l. Afterwards, the patient developed recurrent foetor alcoholic despite abstaining from alcohol. Home Breathalyzer testing consistently showed elevated levels, prompting referral to a gastroenterologist. In 2023, a SIBO test was positive, furthermore, the stool probe showed positive Proteobacteriae, mainly Enterobacter spp. Klebsiella spp. were found negative. Treatment with a 14-day course of rifaximin and probiotics resulted in a two-year asymptomatic period up until recent happenings.

Upon admission, he was stabilized with supportive care in the intensive care unit. He remained afebrile, inflammatory markers were within normal limits (CRP < 5 mg/l, Leukocytes 7.2x10x9/l), and the antibiotic was discontinued. After rehydration, the patient recovered within a few hours and was discharged with a GCS of 15 and without any neurological deficits. The working hypothesis concluded that the patient had experienced endogenous ethanol intoxication due to ABS; likely exacerbated by antibiotic-induced dysbiosis following the recent treatment with Amoxicillin/Clavulanic acid.

Written informed consent was obtained from the patient, and the study was conducted following the CARE checklist.

## Discussion

Alcohol intoxication is one of the most common intoxication diagnoses in an emergency department [9]. In patients with a history of alcohol overuse, clinicians may prematurely attribute recurrent intoxication to relapse, leading to diagnostic anchoring and, delayed consideration of alternative causes such as ABS [8, 10]. Interestingly, prior alcohol overconsumption may also predispose for ABS, possibly through long-term alterations of the gut microbiota, illustrating its dual role as both a confounder in clinical reasoning and a potential etiological factor [8, 10]. A possible misdiagnosis was suspected due to credible reported alcohol abstinence. Overgrowth of bacteria in ABS can lead to nutrient depletion, including carbohydrates and vitamin B12, resulting in caloric loss and vitamin B12 deficiencies [11]. In our patient, a retrospective review of laboratory results revealed chronic Vitamin B12 deficiency. However, a history of alcohol overconsumption can also cause B12 deficiency, complicating interpretation and potentially delaying consideration of ABS. Paramosthy et al (cit) suggest alcoholism, gastrointestinal surgery or pathology, prolonged antibiotic use, high carbohydrate diet, and diabetes mellitus as a risk factor for ABS [8]. In this case, a positive small intestinal bacterial overgrowth (SIBO) test and the detection of increased Proteobacteriae reinforced the role of dysbiosis in the pathogenesis of ABS, while ABS has been associated with yeast species such as *C. albicans*, Candida glabrata, *C. krusei*, and *S. cerevisiae*, increasing evidence suggest that bacterial species may also contribute to endogenous ethanol production. Recent studies identified alcohol producing stains of *Klebsiella pneumoniae* and other Klebsiella species as pathobionts contributing to ABS [12], underscoring the importance of a detailed gastrointestinal history and microbiome analysis [1, 5, 11]. These findings underscore the importance of gastrointestinal evaluation and microbiome assessment. Notably, the patient experienced an intoxication-like episode despite confirmed alcohol abstinence while on Amoxicillin/Clavulanic acid. Emerging evidence indicates that endogenous ethanol production is not limited to fungal fermentation. In an observational cohort, Hsu et al. [13] demonstrated that gut microbial ethanol metabolism contributes directly to ABS identified bacterial ethanol producing pathways as a relevant mechanism. This suggests that microbial imbalance, possibly triggered or worsened by antibiotics, can reactivate endogenous ethanol production. Previous case reports indicate that antibiotic exposure preceded symptoms in 7 of 16 ABS cases [2]. This case highlights the importance of considering ABS as a differential diagnosis in unexplained alcohol intoxication, particularly when clinical abstinence is credible. Accurate diagnosis requires heightened clinical awareness, microbiological evaluation, and interdisciplinary collaboration. In patients with known ABS, antibiotic therapy should be approached with caution, considering possible relapse of the disease. Broader clinical awareness of ABS is essential to improve outcomes, better understanding of diagnosis and risk factors, and prevent legal and social harms that often accompany this underrecognized condition.
